# Waxy Oleogels for Partial Substitution of Solid Fat in Margarines

**DOI:** 10.3390/gels9090683

**Published:** 2023-08-24

**Authors:** Roman Sobolev, Yuliya Frolova, Varuzhan Sarkisyan, Alla Kochetkova

**Affiliations:** Laboratory of Food Biotechnology and Foods for Special Dietary Uses, Federal State Budgetary Scientific, Institution Federal Research Center of Nutrition, Biotechnology and Food Safety, 109240 Moscow, Russia; sobolevrv@bk.ru (R.S.); y.operarius@yandex.ru (Y.F.); kochetkova@ion.ru (A.K.)

**Keywords:** margarine, solid fat, oleogel, saturated fatty acids, beeswax, hydrocarbon

## Abstract

One of the research directions of oleogels is to study the possibility of their practical application in the food industry as an alternative to solid fats. In this work, the possibility of replacing solid fat in margarine (fat content 82.5%) with oleogels was evaluated. The oleogel content varied from 10 to 50% of the fat phase. The concentration of gelator for which beeswax or wax components (9:1 combination of beeswax and hydrocarbons) were used represented 3% in oleogels. The fatty acid composition of the fat components used, their textural characteristics, and their color were studied. The following physicochemical and rheological properties of margarines were determined: color values, textural and thermal characteristics, and sensory properties. The data obtained were processed using principal component analysis (PCA). Oleogels were characterized by lower textural properties compared to commercial fat (CF), but a lower content of saturated fatty acids. When using oleogels, the color characteristics of the margarines changed insignificantly. A decrease in textural and organoleptic properties was shown when using more than 30% oleogel in the composition of margarines. It was found that an increase in the proportion of oleogel leads to a decrease in the melting enthalpy of margarines. The margarines, depending on the ratio of oleogel in the fat phase, were characterized by a content of saturated fatty acids reduced by 7–35% and increased by a 18–92% level of polyunsaturated fatty acids. Thus, the application of oleogels in margarine technology makes it possible to adjust the fatty acid composition while improving the physicochemical properties.

## 1. Introduction

Fats and oils are the basis of many food product formulations and are also used to improve the nutritional value and properties of these products [1]. Widely used are solid fats, usually containing saturated fatty acids in their composition. According to [2], excess consumption of these critically important substances with long chains (C12–C18) is associated with the risk of cardiovascular disease, probably by increasing low-density lipoprotein (LDL) levels. Meanwhile, the use of polyunsaturated fatty acids (PUFAs) is recommended in the diet as part of the fat components instead of saturated fats to reduce LDL levels [3,4]. In this regard, reducing the intake of fats containing long-chain saturated fatty acids is one of the measures to reduce the risk of cardiovascular disease. According to the World Health Organization’s (WHO) recommendations, saturated fatty acid intake should be reduced to less than <10% of the total daily caloric intake [5]. However, saturated fatty acids play a major role in imparting the necessary organoleptic and textural properties to food products and have not been eliminated. For this purpose, advanced alternatives to solid fats—oleogels—are being developed. The concept of producing oleogels is to convert liquid oil into a solid or semi-solid structure that mimics the physical properties and functions of solid fats, which traditionally contain high amounts of saturated fatty acids [6]. Waxes, in particular beeswax (E901), are commonly used as structuring agents, and various oils, such as sunflower oil characterized by a high content of polyunsaturated fatty acids, are used as the fat phase.

In the last decade, the number of studies related to the study of oleogels has increased [7]. At the same time, some of the available works are devoted to studying the possibility of the practical use of oleogels in food technology as an alternative to solid fats. At present, the possibility of replacing solid fat in products has been applied to various types of food products, for example, confectionery products such as muffins and cookies; meat products such as sausages or burgers; fat products such as margarine, shortenings, and spreads [8,9,10,11,12,13,14], etc. An increasing number of studies on the replacement of solid fats with oleogels indicate the promise of developments in this area. Considering that, according to the studies [15,16], beeswax can exhibit emulsifying properties and act as a stabilizer of emulsion systems, its use in the technology of production of food products of an emulsion nature looks especially promising. The use of wax oleogels in spreads and margarines, which are emulsion products, is actively studied [9,17,18], but the studies in which organoleptic and physical properties are both measured are currently limited. It has been shown that the organoleptic profile of products based on wax oleogels can be characterized by a waxy mouthfeel [19] due to a high melting point and a waxy aroma due to the aromatic components that make up waxes [20,21], which may reduce their overall acceptability in organoleptic evaluation.

Beeswax is known as a multi-component system containing substances of various natures (hydrocarbons, wax esters, free fatty acids, free fatty alcohols) [22] and is characterized by high melting points. According to [20], it has been shown that of all the main components of beeswax, hydrocarbons have the least infusibility. At the same time, the authors of [23] determined that the use of individual components of beeswax (for example, hydrocarbons) as a gelator for oleogels may be impractical due to the reduced physical properties of the formed oleogels compared to oleogels based on the initial beeswax. Moreover, the authors noted that an increase in the proportion of hydrocarbons in the composition of an oleogel structured using combinations of wax components makes it possible to reduce melting temperature, correct their physical properties (for example, increase firmness, elastic modulus), and therefore an approach aimed at adding hydrocarbons can be used to the original beeswax. Hydrocarbons can be obtained by the separation (fractionation) of beeswax into separate fractions using preparative flash chromatography, as previously described [20,24]. Hydrocarbons were chosen as the added components due to their low melting temperature, ease of fractionation, and gelling properties. It is assumed that the use of oleogels structured with the presented components will make it possible to obtain products based on them, similar in properties to products using traditional fats.

Thus, the technological task was to develop a product, the traditional composition of which is characterized by a high content of solid fats in emulsified form. Margarines can act as such a product. Margarine is an emulsion product that contains two phases: fat (including oleogels) and aqueous mixed mechanically by the homogenization process [25], which promotes the formation of fine droplets, as well as by using surfactants to reduce the interfacial tension.

The purpose of this study was to examine the properties of margarine with the replacement of solid fat in their composition with oleogels structured with original beeswax or its combination with hydrocarbons.

## 2. Results and Discussion

### 2.1. Fat Component Analysis

In this work, the appearance, color characteristics, fatty acid composition, and textural properties were defined as the evaluated parameters of the fat components used.

#### 2.1.1. Appearance and Color Characteristics

**Figure 1:**
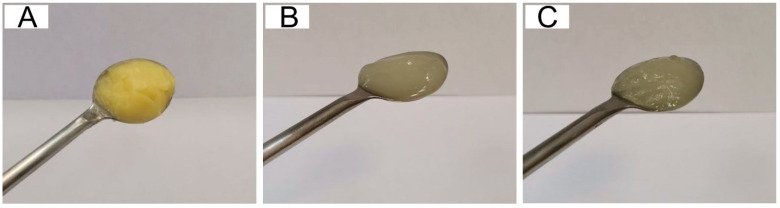
Appearance of fat components: (**A**)—commercial fat (CF), (**B**)—oleogel based on beeswax (BO), (**C**)—oleogel based on combination of beeswax and hydrocarbon (BHO).

The appearance of the commercial fat component and the oleogels produced is shown in Figure 1.

As can be seen from Figure 1, visually, the sample of the CF was characterized by a yellow tint (due to the beta-carotene content), in contrast to the oleogels. The oleogel sample using wax components was more transparent, which may be due to an increase in the proportion of hydrocarbons in the system, which, according to [20], are characterized by a transparent white color.

**Figure 2:**
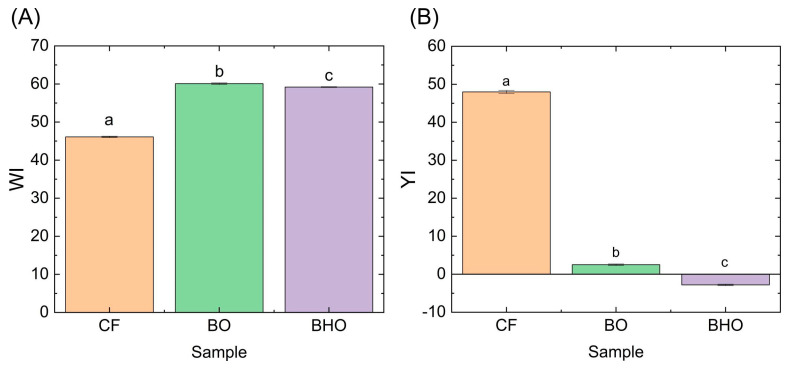
(**A**) Whiteness (WI) and (**B**) yellowness (YI) indices of fat components. Different letters indicate samples differing from each other (*p* < 0.05).

In order to confirm the color differences among the fat components, spectrophotometric color measurement was conducted. The results of color determination are shown in Figure 2.

The whiteness (WI) and yellowness (YI) indices were calculated as part of the color determination. The results indicate that the CF sample had lower WI values but significantly higher YI values. Notably, the oleogel samples were characterized by large WI values and either minimal (for BO) or negative (for BHO) YI values. The difference between the colors value of the oleogels used was statistically significant. Color differences in fat components may further affect the color characteristics of products in which they are planned to be used.

#### 2.1.2. Fatty Acid Composition

The composition of fatty acids (Table S1, Supplementary Materials) of the investigated objects was determined, including commercial fat (CF) component, oleogel on beeswax (BO), and oleogel on beeswax and hydrocarbon combination (BHO). According to the findings, all fat components contained saturated fatty acids, predominantly long chains (C12–C18). However, oleogel samples were characterized by a significantly lower amount of saturated fatty acids (SFAs) relative to CF. The amount of SFAs was equivalent to 42.8% of the sum of fatty acids in CF, and for oleogels 12.5% and 12.4%, respectively. Oleogels were characterized by increased content of polyunsaturated fatty acids (PUFAs). Thus, the PUFA content was equal to 65.9% in the BO and 66.0% in the BHO, with 23.2% in the CF sample. The oleogels were characterized by lower amounts of monounsaturated fatty acids (MUFAs), 21.4% in the BO sample, 21.5% in the BHO sample, and 34.0% in the CF sample. The major fatty acids in the CF were (in descending order): palmitic acid (34.9%—percent of total fatty acids), oleic acid (32.9%), linoleic acid (22.7%), and stearic acid (4.1%). For BO and BHO: linoleic (65.0% and 65.1%, respectively), oleic (20.5% and 20.5%), palmitic (7.5% and 7.4%), and stearic (3.4% and 3.4%). No significant differences were found between the fatty acid compositions among the oleogel samples, so the mean value was used for further calculations.

#### 2.1.3. Texture Properties

At this stage, the textural properties of the fatty components were evaluated: CF, BO, and BHO. The results of texture analysis are presented in Table 1.

According to the obtained data, a significant (*p* < 0.05) difference between the textural properties of the commercial fat component and oleogels was observed. The firmness of the CF one was 2.01 ± 0.07 N, and among oleogels, this index was significantly lower: 0.04 ± 0.01 N for BO and 0.31 ± 0.06 N for BHO. A similar dependence was found for the elastic modulus. Thus, the elastic modulus of CF was 81.72 ± 1.55 N/mm^2^, 2.05 ± 1.30 N/mm^2^ for BO, and 6.39 ± 1.95 N/mm^2^ for BHO. Interestingly, the technique associated with increasing the proportion of hydrocarbons tends to an increase in the textural characteristics of the oleogels. Similar techniques for a successful combination of several wax gelators have been reported previously [9,23,26].

### 2.2. Preparation and Analysis of Margarines

#### 2.2.1. Development of Fat Product Technology

Margarine was chosen as the model product. In our study, the mass ratio of fat in the product was 82.5%. The margarine was prepared using CF as a source of solid fats and represented the basic formulation (control). This fat base was used for comparison due to its high saturated fat content (~43%). A margarine sample using 30% unstructured sunflower oil was also prepared, which corresponded to average values. The submitted sample rapidly became soft and lost its shape at room temperature, making further comparison with other margarines impractical. As test samples, margarines were developed using from 10% to 50% oleogel in the fat phase composition with a gelator concentration of 3%, chosen based on a study [9]. These were samples using oleogel on beeswax (B10–B50) and a combination of beeswax and hydrocarbons (BH10–BH50) in a ratio of 9:1, respectively. This combination was selected to increase the proportion of hydrocarbons in the system based on the results demonstrated earlier [23]. Margarines were prepared according to the technology described in Section 4.4.

The recipes shown in Table 2 were used for production.

Considering that the fat content of the margarine is 82.5%, the recommended serving corresponding to 100 kcal would contain 35–172 mg of beeswax, depending on the oleogel amount. According to JECFA (Joint FAO/WHO Expert Committee on Food Additives), a daily intake of less than 650 mg of beeswax (E901) has no adverse effects for humans [27].

#### 2.2.2. Comparison of Fatty Acid Composition (FAC) of Margarines

Considering that there are no differences among the fatty acid composition of oleogels, margarine samples with the same oleogel content are combined in one column, e.g., B10 and BH10. The differences among the main fatty acid groups of the samples are shown in Table 3.

Thus, according to the data obtained, it is obvious that, with an increase in the oleogel proportion, the content of SFAs decreases by 7.1% at 10% oleogel content and by 35.5% at 50% replacement with oleogels. At the same time, the content of PUFAs increases by 18.4% with a 10% replacement and by 92.2% with the use of 50% oleogels as a replacement for solid fats. The MUFAs content slightly decreased by 3.7% at 10% replacement with oleogels, and by 18.4% at 50% replacement. MUFA reduction can be avoided, for example, by using high oleic sunflower and olive oils. As part of margarines, oleogels based on other oils and their combinations can also be used. At the same time, it should be taken into account that, based on different oils [28,29] and their combinations [30], oleogels with different properties are formed. Various properties in turn can affect the technological margarine characteristics.

Replacing the commercial fat component with oleogels in margarines results in higher PUFA/SFA and to a lesser extent MUFA/SFA ratios (Table 3), which may increase these ratios in the human diet. Increasing these ratios in the diet may lead to reduction in low-density lipoprotein and total to high-density lipoprotein ratios, as well as a reduction in the risks of cardiovascular disease [31].

#### 2.2.3. Visual Assessment

**Figure 3:**
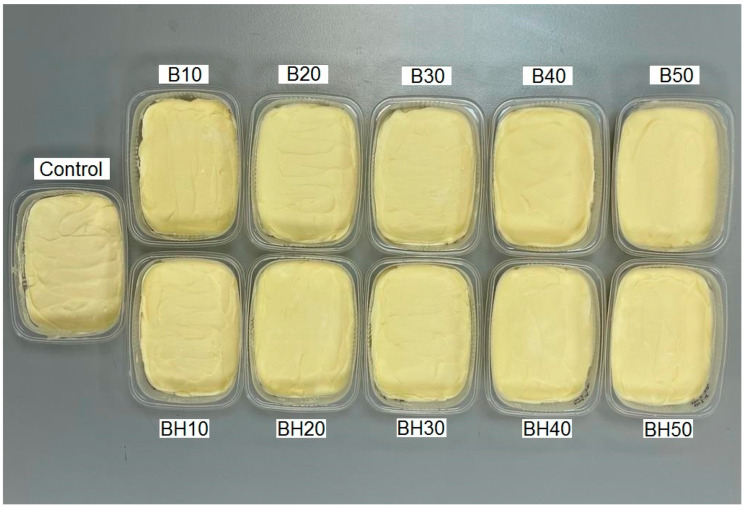
Developed margarine samples. The samples with the basic formulation are designated as “control”, samples “B10–B50” represent margarines in which from 10 to 50% of the fat phase is replaced by oleogels structured with beeswax, and in samples “BH10–BH50”, from 10 to 50% of the fat phase is replaced by oleogels structured with wax components (a mixture of beeswax and hydrocarbons).

Figure 3 shows the appearance of the obtained margarines.

Appearance is the first indicator when making a purchasing decision [32]. According to Figure 3, all samples had a homogeneous surface with no delaminations. The appearance of the margarines was characterized by a light yellow tint, due to the CF included in their composition (Figure 1A and Figure 2).

#### 2.2.4. Color Analysis

The next step was to quantify the color characteristics of the samples using a handheld spectrophotometer. The study of color parameters is important because it is one of the desirable sensory characteristics required by consumers [33]. The color characteristics of the studied margarine samples are presented in Table 4.

According to the data obtained, it can be seen that there are some differences in color among the samples, which is due to the change in the fat phase to oleogels structured with different gelators. The L* lightness values for all the samples ranged from 80.23 ± 0.06 to 85.80 ± 0.26. The highest lightness was characteristic of the sample with the control formulation as well as the BH10 sample. According to [34], a high lightness value is preferable because it does not interfere with the perception of food color. It is shown that, as the portion of oleogel in the final product increases, there is a decrease in the lightness index. A similar trend was observed for indicator (a*), which varied from 1.30 ± 0.00 to 2.77 ± 0.15. Indicator (b*) did not have a similar relationship and ranged from 21.13 ± 0.06 to 23.73 ± 0.35. The lowest value of indices (L*), (a*), and (b*) was characteristic of samples with an oleogel ratio equal to 50% in both sample B50 and sample BH50.

Based on the results obtained, the WI and YI indices were calculated. The whiteness index was similar for all samples and ranged from 26.54 ± 0.58 to 28.98 ± 0.16. According to this indicator, only the B10 and BH20 samples did not differ from the control. Despite significant differences in the yellowness index of the fat components used (Figure 2), in margarines this indicator varied slightly from 36.62 ± 0.91 to 40.20 ± 0.05 (according to Table 4). According to the YI index, only samples B10, B50, BH20, and BH50 did not differ from the control. Despite some differences, all samples had close values of the measured parameters. The WI and YI indicators can likely be more pronounced with a greater replacement of CF by oleogel.

#### 2.2.5. Texture Analysis

Textural properties represent one of the most relevant indicators of food products, and when it comes to fatty products such as margarine, butter, spreads, or shortenings, the main texture parameter is firmness [35]. Firmness refers to the maximum force during indenter penetration. In addition to the firmness parameter, the elastic modulus parameter is evaluated. The results of the textural properties of margarines are shown in Table 5.

According to the data obtained, it was found that the margarine samples differed from each other in their textural properties. The firmness of the samples differed significantly, ranging from 8.32 ± 1.33 N to 33.71 ± 10.10 N. The margarines containing no more than 30% oleogel in their composition were the closest in firmness to the sample with the basic formulation (control) instead of solid fat, with no significant differences. Samples with 40% and 50% oleogel content (B40, B50, BH40, and BH50) had lower firmness and elastic modulus values relative to all samples. The elastic modulus values ranged from 0.36 ± 0.01 N/mm^2^ to 2.81 ± 0.25 N/mm^2^. In terms of modulus of elasticity, the samples with oleogel content equal to 20% (B20 and BH20) had the highest values of modulus of elasticity, which characterized them as more ductile. On the contrary, the samples with higher oleogel contents of 40% and 50% were characterized by lower plasticity. The sample BH30 structured with a combination of wax components (initial beeswax and hydrocarbons) was the closest in terms of elastic modulus value to the basic formulation. According to the textural properties of the fat components (Table 1), the oleogel on wax components had firmness more than two times higher than the oleogel on beeswax components. At the same time, no significant difference was found in the texture of the margarines using these oleogels. A similar result was found in a study [9] which found that it is not the firmness of the oleogels used but the nature of the gelators and their final concentration in the system that makes a higher contribution. The authors also suggest that changes in the textural properties of margarines may be affected by minor components in their formulation, such as monoglycerides, lecithin, and others, which affect the crystallization of the gelator in different ways. Even though the value of elastic modulus in CF is several tens of times higher than in oleogels (Table 1), some samples of margarines with the use of oleogels (mainly samples with oleogel content of 10 and 20%) had higher values for this index. According to available literature data, the improved textural properties of emulsion systems containing waxes may be due to both the combination of Pickering emulsion formation and network stabilization [36] and the consequence of crystal distribution in the continuous phase [15].

#### 2.2.6. Thermal Properties

**Figure 4:**
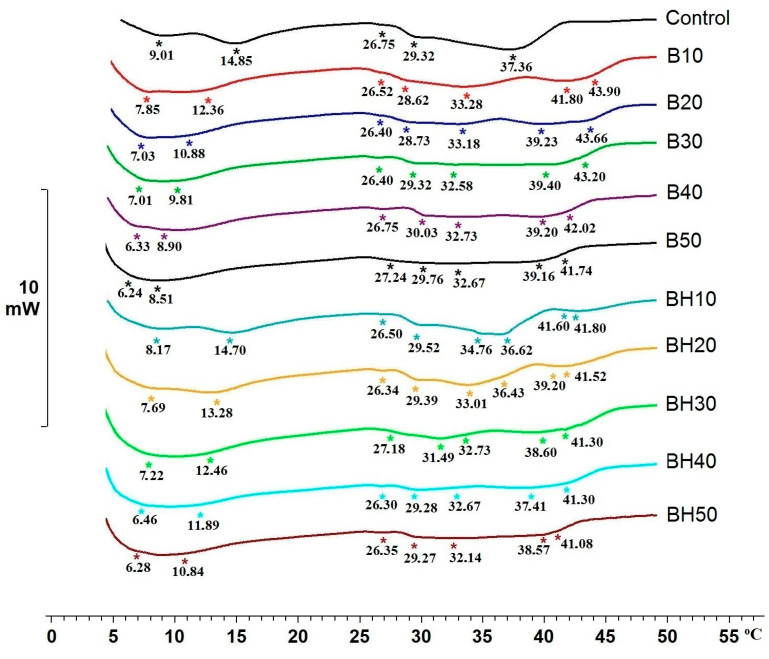
Thermogram of margarine melting. The symbol (*) denotes the melting peak.

The use of margarine formulations with the addition of oleogel with increased hydrocarbon content (BHO) implies a lower melting point due to their lower melting point compared to beeswax [20,23]. The melting characteristics of fatty products, including margarines and spreads, are relevant for flavor disclosure and, consequently, for better product acceptance by the consumer [37]. The obtained results of the thermal characterization of margarines are shown in Figure 4.

**Figure 5:**
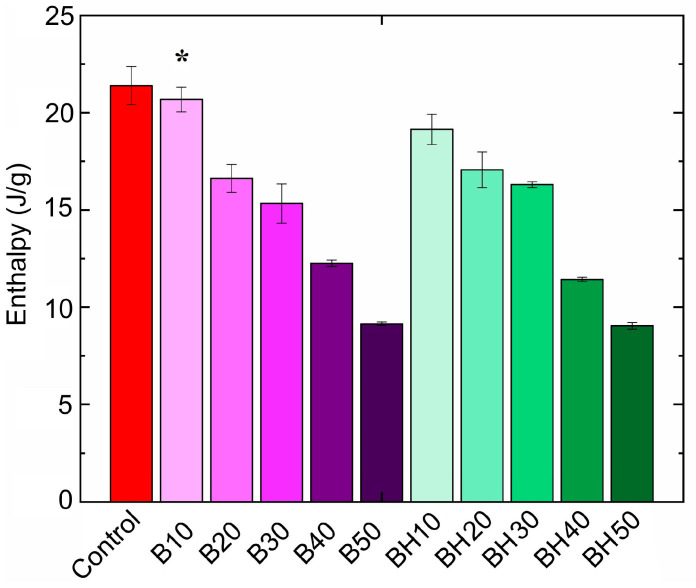
Enthalpy of margarine melting. The symbol (*) denotes the sample with no significant differences from the control sample (*p* > 0.05).

According to Figure 4, it can be seen that several melting peaks (5–8 peaks) were observed in all margarine samples, which indicates the multi-component nature of the system. The control sample was characterized by the least number of peaks (five peaks); in margarines with the presence of oleogels, the number of peaks is higher (from seven to eight), which is due to the components that make up the beeswax. The lowest melting peaks in the control sample were peaks at 9.01 °C and 14.85 °C, which decreased to 6.24 °C and 8.51 °C in the margarine with 50% BO and to 6.28 °C and 10.84 °C with 50% BHO as the proportion of oleogels increased. There were also two weakly pronounced peaks at 26.75 °C and 29.32 °C in the control sample, which were similar to the peaks in this range in all samples. The most pronounced peak in the control sample was in the region of 37.36 °C. Interestingly, with increasing oleogel content (from 10 to 50%), this peak shifts toward lower temperatures. At the same time, the peak characteristics of wax components with higher melting points appear. Therefore, for example, in the B10 sample, these are the melting peaks of 41.80 °C and 43.90 °C, and in the BH10 sample, these are the peaks of 41.60 °C and 41.80 °C, shown in Figure 4. With the highest oleogel content, these peaks are shifted toward lower temperatures to 39.16 °C and 41.74 °C for the B50 sample and up to 38.57 °C and 41.08 °C for the BH50 sample. Therefore, it was found that samples with a higher proportion of hydrocarbons (BH10-BH50) were characterized by lower melting temperatures of the peaks associated with waxy components. This is explained by the fact that the composition of margarines (BH10-BH50) contained a larger amount of low-melting fraction of hydrocarbons, which led to a decrease in the melting temperature of some melting peaks in the system. Along with the melting points, the enthalpy (∆H) was evaluated, as shown in Figure 5.

According to Figure 5, with an increase in the proportion of oleogels, the enthalpy of melting decreased. This is related to the fact that we substituted solid fats (SFAs), which usually have high enthalpy of melting, with oleogels, the enthalpy of fusion of which is much lower due to the presence of a large amount of liquid oil, which does not have high thermal effects. The highest melting enthalpy had a control sample, characterized by the highest saturated (solid) fats content. Samples with the maximum oleogel content of 50% (B50 and BH50) were characterized by the lowest enthalpy values. In [38], a trend toward a decrease in the enthalpy of melting of the fat product (chocolate) was also observed with an increase in the proportion of oleogel. According to [39], the decrease in the enthalpy of melting of the samples is a consequence of the dilution of the system due to an increase in the proportion of oleogel, which indicates the presence of a smaller amount of crystals and crystalline aggregates in these systems.

It has been established that, in our work, the values of the enthalpy of margarines have a close positive correlation with firmness indicators (r^2^ = 0.83). Thus, firmer samples were characterized by higher enthalpy values. Thus, the data obtained indicate the possibility of changing the melting temperatures and enthalpy of margarines depending on the fat phase used. It is supposed that reduced enthalpy values, in turn, can affect the organoleptic properties of the samples; for example, the consistency of the product.

#### 2.2.7. Sensory Analysis

When evaluating the organoleptic characteristics of products based on oleogels structured with waxes, the presence of a taste and aroma characteristic of wax is often noted, which can be a significant obstacle limiting their development [19]. According to the available literature, different effects on organoleptic performance have been observed when wax oleogels were incorporated into other types of products. For example, an article [10] showed that the use of oleogels in fermented sausages resulted in control samples having greater acceptability. Meanwhile, in the study [40], when oleogels were used in bologna sausage, it was shown that the developed products had good overall acceptability. Using oleogels in the composition of biscuits, it is also shown that samples using oleogels can have similar properties to the control sample [41].

**Figure 6:**
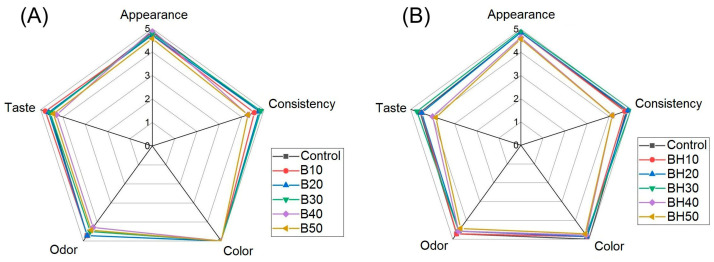
Radar chart of organoleptic evaluation of margarines: (**A**) using BO, (**B**) using BHO.

At the final stage, an organoleptic analysis of the developed margarines was carried out to identify differences among the characteristics depending on the fat phase used. “Taste”, “Odor”, “Consistency”, “Appearance”, and “Color” were used as evaluation indicators, which were evaluated by the expert group on a five-point scale. As a result of the experiment, a radar chart of the estimated parameters was compiled, as shown in Figure 6.

According to Figure 6, the highest values of “Appearance” was characterized by several samples of margarines, including Control, BH30, and B40. The lowest marks were assigned to samples with oleogel content equal to 50%—B50 and BH50. Reduced textural properties of margarines containing 40 and 50% oleogel (Table 5) were reflected in lower scores of the “Consistency”. This indicator was one of the most diverse among the others. The maximum marks of this indicator were exhibited by sample BH30 using 30% BHO. It was found that, when the oleogel content increased to 40 and 50% in samples B40 and B50 as well as BH40 and BH50, there was a decrease in the “Consistency” of the product. It was observed by the expert panel that these samples were softer than the others. This may be because these samples have a higher proportion of fat components with lower saturated (solid) fat content and are characterized by lower firmness and elastic modulus values as described above, as well as a lower enthalpy of melting. According to [9], a higher concentration of the gelator can be used to change the consistency and enhance the textural properties. However, this higher concentration is likely accompanied by a waxy mouthfeel when waxes are used as gelators. This study revealed significant differences in the Flavor indicator. The highest values were characterized by samples B10 and BH30, outperforming the Control. Sample BH30 was more often noted by the experts as the most liked. Nevertheless, all samples with oleogel content over 30% had slightly lower “Taste” scores, especially for samples with wax components (BH40 and BH50). Some experts noted that samples with oleogel contents of 40% or more (B40 and B50 as well as BH40 and BH50) had a waxy mouthfeel. This is because the proportion of wax in these systems increases, thereby giving the product an extraneous waxy mouthfeel. Given the reduced enthalpy values of these samples, but the presence of waxy mouthfeel, it can be assumed that they do not have a direct relationship with each other. Thus, the perception threshold of waxy components in margarines with this formulation feels at their content of slightly less than 1% of the total mass of components included in the formulation. Another factor contributing to the lower taste scores of margarines with 40% or more oleogel content could have been a change in the fatty acid profile. As described earlier (Section 2.1.2), the main fatty acid of the commercial fat component used in the Control margarine formulation was palmitic acid (saturated), while the oleogels (BO, BHO) used in the experimental margarine formulations were dominated by linoleic acid (unsaturated). The obtained data are consistent with the study [42] in which it was shown that the taste and flavor of some products using margarines deteriorates with increasing unsaturated fatty acids, especially polyunsaturated fatty acids.

The sample with the basic formulation and samples B10-B50 showed the highest values of the “Color” indicator. A slight decrease in “Color” scores in samples BH10-BH50 may be because the oleogel used with a higher content of hydrocarbons has a more transparent color (Figure 1), while according to the definition of “Color” of margarines using a spectrophotometer, no significant differences between margarine samples were found. Most experts noted that margarine samples with oleogel contents over 30% (samples B40, B50, BH40, BH50) had a shinier surface.

When evaluating the parameter “Odor” in samples with up to 30% oleogel on beeswax (B10-B30) and up to 40% on combination of beeswax and hydrocarbons (BH10-BH40), no reliable differences from the control sample were found, which indicates the absence of a negative influence of aromatic compounds from beeswax and hydrocarbons at this concentration. At the same time, samples with a higher proportion of oleogels—B40, B50, and BH50—were evaluated lower than the others, which may be due to the manifestation of ongoing changes in the aromatic profile of margarines caused by compounds present in the composition of beeswax or its components [20].

**Figure 7:**
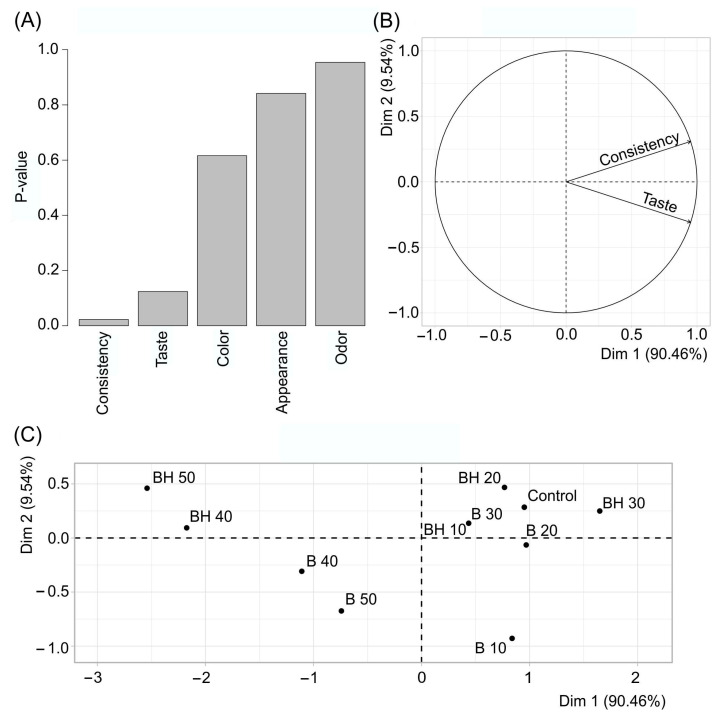
PCA analysis of margarines: (**A**)—reliability of differences in descriptors, (**B**,**C**)—variation between samples.

Based on the results of the organoleptic evaluation, a principal component analysis (PCA) was performed (Figure 7).

It was determined that, among all the descriptors studied, texture and taste differed the most between samples (Figure 7A), so the variation between samples was studied only based on differences in these descriptors (Figure 7B,C). The overall variability explained for organoleptic properties based on the first two significant measurements was 100% (since there are only two factors), with Dim1 and Dim2 accounting for 99.46% and 9.54%, respectively (Figure 7B). The first dimension is positively correlated with both “Consistency” and “Taste”. The second dimension correlates positively with “Consistency” and negatively with “Taste”. It is characteristic that samples with a high content of hydrocarbons are characterized by the lowest taste score (average 4.000 and 3.857 for BH40 and BH50, respectively), and samples with a high beeswax content—the lowest estimate of the spreadable consistency equal to 4.143 for B40 and B50. Sample BH30 had the highest score for both consistency (5.000) and taste (4.714), exceeding the score for the control sample (4.857 and 4.571, respectively).

As a result of this work, it has been revealed that it is recommended to use no more than 30% oleogel (with 3% of beeswax and hydrocarbons combination) as a fat base to produce margarines with the presented formulation. Margarines with a reduced content of saturated fatty acids and increased content of polyunsaturated fatty acids but with similar physical and organoleptic characteristics with the margarine prepared with the use of the CF were developed as a result of the use of oleogels. 

## 3. Conclusions

In this work, the use of oleogels based on beeswax and its combination with hydrocarbons as an alternative to solid fat (replacement 10–50%) in the margarines was studied. As a result of the analysis of the composition of fatty acids, it was shown that the content of saturated fatty acids in oleogels is more than three times less than the commercial fat component. It has been determined that the firmness and elastic modulus of the commercial fat component are significantly higher compared to the presented oleogels. We found that, in terms of firmness, all margarine with an oleogel content of up to 30% did not have significant differences from the control but differed in elasticity modulus. When evaluating the color characteristics of margarines, it was found that the use of oleogels did not lead to a significant change in the studied indicators. It was found that the use of oleogels leads to an increase in the number of melting peaks, while an increase in the proportion of oleogels in the system leads to a decrease in the melting enthalpy. When determining the sensorial properties, it was found that the margarine using 30% oleogel in combination with beeswax and hydrocarbons was the most preferable. The use of formulations with 30% oleogel will reduce the content of saturated fatty acids by 21% and increase the content of polyunsaturated fatty acids by 55%. Thus, the prospects of using oleogels in the formulation of margarines as an alternative to solid fats with a reduced content of saturated fatty acids have been confirmed. 

## 4. Materials and Methods

### 4.1. Materials

Refined deodorized sunflower oil “Sloboda” (EFKO, Alekseevka, Russia), beeswax (Dom Voska, Nizhny Novgorod, Russia), and a fraction of hydrocarbons (>99%) obtained from beeswax by fractionation, according to the method in [23], were used for oleogel preparation.

Commercial fat component “Ecolakt 1403-33” (EFKO, Alekseevka, Russia), which is based on fractionated and partially hydrogenated vegetable oils and fats, distilled monoglycerides DMG 0291 (Palsgaard, Denmark), soy lecithin Adleck (ADM, The Netherlands), cream flavoring (MacomRus, Moscow, Russia), salt (Company MS, Moscow, Russia), sorbic acid (Corn product, Moscow, Russia), and citric acid (Dr. Oetker, Belgorod, Russia) was used for margarine development.

Hexane, acetone (Component-Reactive, Moscow, Russia), methanol, stabilized chloroform, acetyl chloride (Sigma-Aldrich, St. Louis, MO, USA), standard mixture FAME 37 Component mix in dichloromethane (Bellefonte, PA, USA), and undecanoic acid standard (C11-ME) (Bellefonte, PA, USA) of analytical grade were used for chromatography.

### 4.2. Oleogel Preparation

The gelator concentration in the oleogels, equal to 3%, was chosen based on the study [9] in which oleogels were used as a fat component in the production of margarines. Sunflower oil was used as the oil phase. The initial beeswax or combination of beeswax and hydrocarbons in the ratio (9:1) was used as a gelator. To prepare oleogels, sunflower oil in a glass beaker was heated on a stove to a temperature of 90 °C; then, a gelator was added and the contents of the glass were mixed on a magnetic stirrer at a rate of 300 rpm for 20 min until complete melting. The samples were cooled to a temperature of 20 °C at a rate of 1 °C/min, after which they were stored for 24 h for further research and use in the margarine formulation.

### 4.3. Analysis of Fat Components

The color, fatty acid composition, and textural properties were analyzed.

#### 4.3.1. Color Analysis 

The color of fat components was measured using a portable colorimeter BS 7016 (3NH, China). The International Commission on Illumination (CIE) recommends using the system (L*a*b*), as well as whiteness (WI) and yellowness (YI) indices for color evaluation. Thus, the measured and calculated parameters were as follows: L*—lightness (varies from 1 to 100, i.e., from the darkest to the lightest), a*—color position in the range from green to magenta, and b*—from blue to yellow. The CIE L*a*b* color scale is a standard scale for evaluating the lightness index (L*), which ranges from black (0) to white (100). The parameter (a*) ranges from green (negative values) to red (positive values), while the component (b*) ranges from blue (negative values) to yellow (positive values). The values of L*, a*, and b* were automatically calculated using the instrument software. Measurements were performed at four different points of the fat component. Based on the results obtained, the whiteness and yellowness indices were calculated. The whiteness index (WI) was calculated according to [43] using Equation (1):

(1) $$WI=\sqrt{{\left(100-L^{*}\right)}^{2}+a^{*2}+b^{*2}}$$

The yellowness index (YI) was calculated according to the research [44]. To calculate the YI, we used Equation (2):

(2) $$YI=142.86\frac{b^{*}}{L^{*}}$$

#### 4.3.2. Analysis of Fatty Acid Composition

Fatty acid composition of fat components was analyzed using an Agilent Technologies 7683B Series gas chromatograph (Agilent Technologies, Santa Clara, CA, USA) with a flame ionization detector on a 100 m Agilent J&W GC Columns Select FAME (Agilent Technologies, Amstelveen, The Netherlands), 25 mm × 0.25 μm [28,45]. Samples (10–15 mg) were placed in a 5 mL tube, and then 2 mL of methanol, 20 μL of chloroform, and 2 μL of acetyl chloride were added. The tube was sealed tightly with a lid and spacer and placed in a thermostat FED 53 (Binder, Tuttlingen, Germany) at 80 °C for 1 h. After cooling (about 10 min), 2.5 mL of hexane and 70 μL of distilled water were added to the samples. The samples were covered again and stirred on a vortex for about 30 s. The samples were allowed to stand for about 5 min, and then 1 mL of the upper phase was transferred into the vial for analysis. The injection volume was 1 μL, the mode was 30:1 flow division, the carrier gas was nitrogen, and the flow rate was 0.9 mL/min. The injector temperature was 260 °C and detector temperature was 240 °C. 

The separation conditions were as follows: initial temperature of 140 °C (isotherm for 5 min) increased at a rate of 4 °C/min to 220 °C, isotherm 25 min. Data were collected and processed using Agilent ChemStation Rev.B.04.03 software (Agilent Technologies, Santa Clara, CA, USA).

#### 4.3.3. Texture Analysis

Analysis of the textural properties of fatty components (CF, BO, BHO) was performed using the universal testing machine EZ-test-SX (Shimadzu Corporation, Suzhou Instruments Manufactureing, Suzhou, Jiangsu, China). The samples were kept for 24 h at 20 °C before testing. A cylindrical nozzle (12 mm diameter, Perspex) was used as an indenter. The rate of immersion of the indenter into the sample was 10 mm/min and the penetration depth was 10 mm (50% of sample height). Firmness and elastic modulus were measured automatically using Trapezium X software (Shimadzu, Japan).

### 4.4. Preparation and Analysis of Margarines

The margarines were prepared using the methodology described previously [9] with the modification of recrystallization of the fats. In the first stage, the oil phase was prepared by heating the pre-prepared oleogel in a glass beaker in a water bath heated to 80 °C, to which the remaining components of the fat phase were subsequently added: commercial fat (CF), soy lecithin, and monoglycerides, constantly mixing with a magnetic stirrer at speed of about 300 rpm. The water phase was prepared by adding salt, sorbic acid, skimmed milk powder, flavor, and citric acid to a glass beaker with water kept in a water bath at 80 °C with constant mixing using a magnetic stirrer at a speed of 300 rpm. The next step was to gradually add the water phase to the fat phase. When mixing, the temperature of the fat and aqueous phases should be in the range of 60–65 °C and the mixture is stirred on a homogenizer Silent Crusher M (Heidolph, Schwabach, Germany) at a speed of about 5000 ± 100 rpm for 5 min. As is known [46], fat crystallization is the key process affecting the structure and properties of fat-based products, including margarines. Therefore, the obtained samples were further cooled in an ice bath, intensively stirring until solidification. According to [17], the quenching approach allows for a reduction in crystal size, therefore improving the perception of the margarine in the mouth, but this will lead to a reduction in textural characteristics compared to more gradual cooling. Another step was to carry out the recrystallization process, for which samples were first kept for 24 h in a refrigerator at a temperature of 4 °C, then placed in a freezer at −18 °C for 48 h, after which they were returned to the refrigerator for 12 h.

#### 4.4.1. Color Analysis

The color of margarines was measured similarly to the previously described methodology for determining the color of fat components, as seen in Section 4.3.1. 

#### 4.4.2. Texture Analysis

Textural properties of margarines were determined on a Shimadzu EZ-test-SX universal testing machine using a cylindrical nozzle (12 mm diameter, Perspex) by penetrating 10 mm inside a 20 mm thick sample at a speed of 10 mm/min. Firmness and modulus of elasticity were measured automatically using Trapezium X software (Shimadzu, Japan).

#### 4.4.3. Differential Scanning Calorimetry (DSC)

Melting temperatures and enthalpy of margarines were measured using a differential-scanning calorimeter DSC 3 (Mettler-Toledo, Greifensee, Switzerland). For analysis, a suspension containing 8–10 mg of margarine was placed in an aluminum crucible and hermetically sealed. The measurement cycle included three sections: cooling, stabilization, and heating. In the first step, the samples were cooled to 4 °C at a rate of 10 °C/min, then stabilized at this temperature for 5 min and heated to 100 °C at a rate of 7 °C/min. The data obtained were processed in the STARe program (Mettler-Toledo).

#### 4.4.4. Sensory Evaluation

Organoleptic properties of processed margarines were evaluated using the criteria “Consistency”, “Appearance”, “Color”, “Odor”, and “Taste”. The evaluation was performed using a 5-point hedonic scale [42]. In the first stage of research, seven experts with at least 5 years of experience—two men and five women (28–70 years old)—were selected according to their threshold for basic tastes, odor, and color identification [47]. The evaluation was performed in individual specialized booths designed in compliance with the ISO guidelines [48]. The total number of 11 margarine samples were analyzed. The concordance between the panelists was ensured by training all assessors together and by the same panel leader. Panel training was performed in two sessions, each lasting 3–4 h, over a period of 2 days prior to the sensory analysis. The panelists were given unsalted crackers and drinking water (22 ± 1 °C) to cleanse their palates to reduce the impact of the precedence effect. The intensity of each attribute was rated using the 5-point scale (1—absence of the sensation and 5—maximum intensity). The descriptive analysis of the margarine was replicated three times with a fresh batch of margarine each time. 

### 4.5. Statistical Analysis

Graphical images were produced using OriginPro 2018 software. One-way analysis of variance (ANOVA) was used to assess the variation in the groups followed by Tukey’s post hoc test. The significance level was *p* < 0.05 with a 95% confidence level. 

For analysis of the sensory data R software, version 4.3.1 was used with the ‘‘SensoMineR’’ package [49].

## Figures and Tables

**Table 1:** Textural properties of fat components.

Sample	Firmness (N)	Elastic Modulus, N/mm^2^
CF	2.01 ± 0.07 ^a^	81.72 ± 1.55 ^a^
BO	0.04 ± 0.01 ^b^	2.05 ± 1.30 ^b^
BHO	0.31 ± 0.06 ^b^	6.39 ± 1.95 ^c^

Different letters indicate samples differing from each other (*p* < 0.05).

**Table 2:** Margarine recipes.

Sample		Fat Phase 82.5%					Aqueous Phase 17.5%					
	CF	CF Replacement			MG	SL	Water	SMP	Flavoring	Salt	SA	CA
		SO	BW	HC								
Control	81.90	-	-	-	0.50	0.10	14.96	2.00	0.30	0.15	0.07	0.02
B10	73.71	7.94	0.25	-								
B20	65.52	15.89	0.49	-								
B30	57.33	23.83	0.74	-								
B40	49.14	31.78	0.98	-								
B50	40.95	39.72	1.23	-								
BH10	73.71	7.94	0.22	0.03								
BH20	65.52	15.89	0.44	0.05								
BH30	57.33	23.83	0.67	0.07								
BH40	49.14	31.78	0.88	0.10								
BH50	40.95	39.72	1.11	0.12								

SO—sunflower oil; BW—beeswax; HC—hydrocarbon; CF—commercial fat; SMP—skimmed milk powder; SL—soy lecithin; MG—monoglycerides; CA—citric acid; SA—sorbic acid.

**Table 3:** Content of different fatty acid groups in margarine samples.

Type of Fatty Acid	Sample					
	Control	B10/BH10	B20/BH20	B30/BH30	B40/BH40	B50/BH50
SFAs, % Difference from control, %	35.3	32.8	30.3	27.8	25.3	22.8
	-	−7.1	−14.2	−21.3	−28.4	−35.5
PUFAs, % Difference from control, %	19.1	22.7	26.2	29.7	33.2	36.8
	-	18,4	36.9	55.3	73.7	92.2
MUFAs, % Difference from control, %	28.0	27.0	26.0	24.9	23.9	22.9
	-	−3.7	−7.4	−11.1	−14.8	−18.4
PUFAs/SFAs	0.5	0.7	0.9	1.1	1.3	1.6
MUFAs/SFAs	0.8	0.8	0.9	0.9	0.9	1.0

SFAs—saturated fatty acids, PUFAs—polyunsaturated fatty acids, MUFAs—monounsaturated fatty acids.

**Table 4:** Color characteristics of margarines.

Sample	L*	a*	b*	WI	YI
Control	85.17 ± 0.67	2.77 ± 0.06	21.83 ± 0.49	26.54 ± 0.58	36.62 ± 0.91
B10	83.73 ± 0.45 *	2.60 ± 0.10	21.70 ± 0.50	27.24 ± 0.60	37.02 ± 0.97
B20	84.50 ± 0.36	2.30 ± 0.00 *	23.67 ± 0.40 *	28.38 ± 0.24 *	40.01 ± 0.57 *
B30	83.03 ± 0.42 *	2.45 ± 0.06 *	23.37 ± 0.12 *	28.98 ± 0.16 *	40.20 ± 0.05 *
B40	82.43 ± 0.31 *	2.13 ± 0.06 *	22.53 ± 0.06	28.65 ± 0.24 *	39.05 ± 0.24 *
B50	81.67 ± 0.06 *	1.43 ± 0.06 *	21.13 ± 0.06	28.01 ± 0.04 *	36.97 ± 0.09
BH10	85.80 ± 0.26	2.77 ± 0.15	23.73 ± 0.35 *	27.80 ± 0.22 *	39.52 ± 0.49 *
BH20	84.30 ± 0.46	2.30 ± 0.17 *	22.23 ± 0.38	27.31 ± 0.53	37.68 ± 0.79
BH30	83.47 ± 0.06 *	2.23 ± 0.06 *	23.27 ± 0.06 *	28.63 ± 0.07 *	39.82 ± 0.12 *
BH40	82.60 ± 0.26 *	1.90 ± 0.00 *	22.47 ± 0.21	28.48 ± 0.33 *	38.86 ± 0.49 *
BH50	80.23 ± 0.06 *	1.30 ± 0.00 *	21.13 ± 0.06	28.97 ± 0.07 *	37.63 ± 0.12

The symbol (*) marks samples that differ from the control (*p* < 0.05).

**Table 5:** Textural properties of margarines.

Sample	Firmness (N)	Elastic Modulus (N/mm^2^)
Control	30.84 ± 9.65	1.12 ± 0.21
B10	27.74 ± 8.25	1.75 ± 0.01 *
B20	28.07 ± 10.32	2.68 ± 0.26 *
B30	20.65 ± 6.12	1.55 ± 0.04 *
B40	10.50 ± 1.94 *	0.36 ± 0.01 *
B50	9.33 ± 2.73 *	0.43 ± 0.05 *
BH10	33.71 ± 10.10	1.86 ± 0.12 *
BH20	26.34 ± 9.45	2.81 ± 0.25 *
BH30	20.49 ± 5.30	1.23 ± 0.16
BH40	11.80 ± 4.56 *	0.58 ± 0.05 *
BH50	8.32 ± 1.33 *	0.68 ± 0.13

The symbol (*) marks samples that differ from the control (*p* < 0.05).

## Data Availability

All data are available in the manuscript and supplementary materials.

## Supplementary Materials

The following supporting information can be downloaded at: https://www.mdpi.com/article/10.3390/gels9090683/s1, Table S1: Fatty acid composition of fat components.
