# Supplementary material for: Waxy Oleogels for Partial Substitution of Solid Fat in Margarines
Source: Gels. 2023 Aug 24;9(9):683. doi: 10.3390/gels9090683 (PMC10530580; doi:10.3390/gels9090683)
Supplement: Supplementary file 1 [file gels-09-00683-s001.zip › gels-2543130-supplementary.pdf]

gels

MDPI

Supplementary materials

Table S1. Fatty acid composition of fat components

Fatty acid index	CF	BO	BHO
**C 6:0**	0.02	-	-
**C 8:0**	0.20	-	-
**C 10:0**	0.17	-	-
**C 12:0**	1.40	-	-
**C 14:0**	1.30	0.09	0.11
**C 15:0**	0.06	-	-
**C 16:0**	34.88	7.50	7.41
**C 16:1**	0.03	-	-
**C 16:1, 9 – *c***	0.15	0.12	0.11
**C 17:0**	0.09	0.04	0.05
**C 17:1**	0.03	-	-
**C 18:0**	4.06	3.41	3.38
**C 18:1, 9 – *t***	0.10	0.06	0.06
**C 18:1, 9 – *c***	32.85	20.48	20.52
**C 18:1, 11 – *t***	0.69	0.68	0.71
**C 18:1, 11 – *c***	0.01	-	-
**C 18:2, 9 – *c*, 12 – *t***	0.19	0.40	0.42
**C 18:2, 9 – *t*, 12 – *c***	0.17	0.35	0.31
**C 18:2**	22.70	65.04	65.09
**C 18:3**	0.13	0.11	0.13
**C 20:0**	0.30	0.16	0.15
**C 20:1**	0.13	0.14	0.14
**C 22:0**	0.22	0.83	0.85
**C 24:0**	0.11	0.48	0.40
**Total**			
**SFA**	42.81^{a}	12.51^{b}	12.35^{b}
**PUFA**	23.19^{a}	65.90^{b}	65.95^{b}
**MUFA**	33.99^{a}	21.42^{b}	21.48^{b}

SFA – saturated fatty acids, PUFA – polyunsaturated fatty acids, MUFA – monounsaturated fatty acids. The letters (a, b) indicate significant differences ($P<0.05$)
